# Educational Intervention on Awareness of Health-Damaging Behaviors in Educators

**DOI:** 10.3390/sports12120348

**Published:** 2024-12-18

**Authors:** Valentina Focaroli, Marina Chiaro, Maria Vittoria Battaglia, Laura Guidetti, Andrea Velardi

**Affiliations:** 1 Department of Economic, Psychological and Communication Sciences, Niccolò Cusano University, 00166 Rome, Italy; 2 Department of Human Science, Link University, 00165 Rome, Italy; m.chiaro@unilink.it; 3 Department of Humanities, Movement and Education Science, Niccolò Cusano University, 00166 Rome, Italy; mariavittoria.battaglia@unicusano.it (M.V.B.); laura.guidetti@unicusano.it (L.G.); andrea.velardi@unicusano.it (A.V.)

**Keywords:** prevention, doping, antidoping

## Abstract

Doping prevention transcends elite sports, highlighting a broader societal challenge where performance enhancement is driven by pressures to increase strength, beauty, and status. This issue extends to adolescents and non-competitive sports participants, where self-optimization pressures are increasingly normalized. Research underscores the need for tailored educational interventions that go beyond punitive measures, fostering ethical decision-making and personal responsibility. The recent literature emphasizes that addressing psychological protective factors, such as self-efficacy and emotional regulation, can effectively mitigate substance use risks. The project “Sport Informa” involved the educational community and adolescents between the ages of 16 and 19, with the goal of providing knowledge about the phenomenon of doping and acquiring tools useful for enhancing self-esteem and self-management skills, in order to prevent the risk of doping by promoting a balanced psychophysical development in young people. A 16 h online training program was delivered to high school teachers. The training provided knowledge about doping, its psychological and social implications, and practical tools for promoting protective factors in students. A longitudinal design was employed, with data collected before and after the intervention using validated psychometric tools, including the Self-Efficacy Scale, the Self-Compassion Scale, and the Emotional Regulation Questionnaire. Results showed significant improvements in teachers’ psychological dimensions, particularly in self-efficacy and emotional regulation, which they subsequently applied in their interactions with students. Finally, a survey was conducted with young participants who took part in the project activities to assess their self-evaluation of key factors relevant to designing future educational interventions and events aimed at preventing doping use.

## 1. Introduction

Doping prevention extends far beyond elite sports, addressing a broader societal challenge. This perspective stems from the recognition that the drive to enhance performance is not confined to athletes but is rooted in a wider social context, where success in sports and physical appearance are often linked to attributes like strength, competence, beauty, and social status [1]. In modern society, characterized by a growing focus on self-improvement and optimization, the desire for physical enhancement transcends athletic domains, influencing diverse populations, including those not traditionally involved in competitive sports [2]. Research has shown that such pressures can contribute to the normalization of performance-enhancing behaviors, leading individuals to resort to substances like anabolic steroids or stimulants to achieve their aspirations (e.g., [3]). Viewing doping through a socio-cultural framework rather than solely as an individual or generalized social issue, Pedersen (2010) [4] found that the use of doping substances and legal performance-enhancing drugs varies significantly across training contexts. In some cases, this variation is shaped by factors such as gender, age, educational background, and training frequency. Notably, Pedersen’s study highlighted that young men with lower levels of education who train frequently are more likely than other groups to experiment with both legal and illegal pharmaceutical substances. Pursuing physical fitness has increasingly led to the consumption of substances aimed at boosting performance and appearance, commonly referred to as performance- and image-enhancing substances (PIES) [5]. Adolescents and young adults, who are in the process of shaping their identity within a culture that heavily emphasizes physical appearance, are likely to be particularly at risk [6]. Other types of substances, called Image and Performance Enhancement Drugs (IPEDs), refer to a broad category of substances used specifically to enhance physical appearance—such as by increasing muscle mass or reducing body fat—and/or to improve physical or athletic performance [7]. The National Survey on IPEDs used in England [8] highlighted that the motivations for using these substances are varied. Among these, altering physique or achieving cosmetic enhancements emerged as a highly significant factor driving the use of them. 

As suggested by Saiphoo and Vahedi (2019) [9], engaging with social media, especially content that emphasizes visuals and physical appearance, has been linked to a decline in perceived body image. Social media contents predominantly reflect society’s constructed appearance ideals, which are heavily focused on thinness and muscularity. These ideals lack diversity, particularly in the representation of gender, abilities, as well as other characteristics. The constant exposure to images portraying these unrealistic appearance standards creates a visual environment on social media that represents only a small fraction of the diversity of body types and appearances found in the general population. This limited representation can lead individuals to become more concerned about their own body image and to seek ways to alter their appearance to align with these unrealistic ideals [10].

Adolescence is a critical period for the development of self-identity and self-esteem, and the desire to enhance one’s physical abilities and appearance is often heightened during this stage of life. In particular, young people demonstrate a growing interest in self-optimization, which can involve substance use, both within and beyond competitive sports [2,11]. Poor self-esteem and a distorted body image are major contributors to the increased risk of anabolic-androgenic steroid (AAS) abuse, frequently driven by the desire to quickly attain a socially idealized physique [12,13]. Additionally, low self-confidence, often reflected in uncertainty about one’s capabilities, can lead individuals to overcompensate by obsessively striving for a perceived perfect appearance, which may result in engaging in harmful practices.

To enable individuals to make informed decisions regarding doping, a robust educational foundation is crucial. Education should not focus solely on the risks and consequences of doping but also aim to cultivate ethical values and personal responsibility. Research has indicated that educational interventions designed to foster ethical decision-making are more effective at preventing doping than punitive measures alone [14]. 

Furthermore, such education must be carefully tailored to the specific needs and circumstances of learners, ensuring it effectively fosters lasting change. This tailoring is crucial because the motives behind doping can vary significantly across different groups—what drives a professional athlete to dope may be different from what motivates an adolescent participating in recreational sports. Research highlights that adolescents often underestimate the health risks associated with performance-enhancing drugs, which further emphasizes the need for comprehensive educational programs [15]. When examining the goals of doping prevention, it becomes clear that preventative measures should be introduced as soon as possible, ideally before doping behaviors can manifest (around 16 years old), in the form of primary prevention. Early prevention not only mitigates the risk of doping but also helps instill values such as fair play, integrity, and respect for oneself and others—principles that can be applied beyond the sports context. Currently, anti-doping initiatives largely target competitive athletes and are mandatory for them (e.g., [16]). These measures typically involve random testing and the enforcement of penalties for violations, but their scope is limited when it comes to addressing non-competitive groups. However, viewing doping prevention as a broader social concern [17] reveals that while competitive athletes may be more directly exposed to doping controls, adolescents in non-competitive settings are often overlooked by current policies and interventions. 

Adolescents’ intentions to engage in doping are significantly shaped by belief systems specifically related to doping, such as holding more favorable attitudes toward its use [18]. This highlights the importance of psychological and attitudinal factors in doping behavior [19]. In this regard, over the past ten years, studies investigating these factors have grown substantially and numerous personal and socio-contextual influences—such as goal achievement orientations, ethical principles, and societal expectations—have been identified as either promoting or discouraging doping. The review of Ntoumanis and colleagues (2014) [20] studied the psychological and social determinants of doping intentions and behaviors in sports and fitness contexts and identified several key influences. Specifically, the strongest risk factors included favorable attitudes toward doping, social norms supporting doping, and the use of dietary supplements. Conversely, a strong level of morality and high self-efficacy were identified as the most effective protective factors against doping. 

The study by Lucidi et al. (2008) [21] measured participants’ moral disengagement, specifically their tendency to detach from the moral implications of their unethical actions. The findings indicated that this variable predicted both doping intentions and behaviors. Barkoukis et al. (2011) [22] found that athletes with a high level of fair play, autonomous motivation (stemming from enjoyment or personal values), and achievement goals focused on personal improvement and effort reported a lower risk of doping compared to those with low levels of fair play, motivation driven by external pressures, social approval, or guilt, and achievement goals centered on performance outcomes (i.e., emphasis on displaying normative superiority). In this line, the metanalysis of Ntoumanis et al. (2014) [20] aimed to study the key psychosocial factors, both positive and negative, influencing doping behaviors and intentions. The research evaluated not only psychological aspects (e.g., attitudes) and social-contextual factors (e.g., societal norms) but also the role of demographic characteristics such as age and gender in predicting doping-related behaviors. The study examined the impact of psychological factors on doping tendencies. It found that athletes with a task-focused approach were less likely to engage in doping, whereas those driven by ego-oriented goals or externally controlled motivations showed a higher likelihood. This suggests that individuals with ego-driven or externally regulated mindsets are more prone to maladaptive behaviors. The study also demonstrated that favorable attitudes toward doping and skewed beliefs about its prevalence and social acceptance significantly increased doping intentions. Conversely, individuals with higher self-control and self-efficacy were less likely to engage in doping. Additionally, the study found that higher levels of sportsmanship and lower moral disengagement were strongly associated with a reduced likelihood of doping. Again, this highlights the critical role of ethical values in deterring doping behavior. Moreover, body image concerns—specifically dissatisfaction with muscularity or thinness—were found to have a significant link to increased doping intentions and use. This suggests that negative perceptions of body image may encourage doping as individuals seek to modify their physique through illegal substances. Consequently, prevention efforts, particularly those aimed at young athletes, should prioritize addressing body image concerns to mitigate the risk of doping. This highlights the importance of extending preventive measures beyond competitive settings and ensuring that all individuals, regardless of their sporting level, are educated about the dangers of doping and the values of clean sport. Fortunately, doping prevention efforts are expanding to include more diverse groups, such as those in recreational and non-competitive sports.

Research shows that a multi-faceted approach, which includes education, peer support, and role models, can have a more profound impact on preventing doping than standalone interventions [23]. Anti-doping agencies are increasingly focusing on schools, offering prevention programs specifically tailored to students. For example, the World Anti-Doping Agency (WADA) has launched initiatives such as the “Sport Values for Every Classroom” project, which aims to integrate anti-doping education into school curricula [24]. However, there is limited data on the implementation and effectiveness of these programs in schools, and more research is needed to evaluate their long-term impact.

Nevertheless, schools—especially through physical education classes—offer a prime setting to engage young people, regardless of their athletic background. The school environment is not only accessible to all adolescents, but it also provides a controlled space where values such as respect, fairness, and integrity can be consistently reinforced. Studies have indicated that school-based interventions can effectively influence attitudes towards doping, especially when coupled with broader discussions about health and well-being [25,26,27,28]. As such, schools are an ideal environment for implementing primary doping prevention, using a cross-disciplinary approach that can effectively address vulnerable adolescent groups in a manner appropriate to their age.

Through broader educational efforts, schools can play a crucial role in fostering awareness and early-stage prevention. A thoughtfully designed educational program, rooted in honesty and respect, could prove more effective in shaping young people’s attitudes towards doping and safeguarding their health. For instance, values-based education, which focuses on developing ethical reasoning and personal responsibility, has been shown to reduce the likelihood of doping behavior [29]. We also know that education about functional daily routines for the individual has positive effects on physical and mental health, as well as overall well-being [30]. 

For these reasons, the Italian “Lotta al Doping” project aimed to initiate a cultural shift in young people by conducting anti-doping seminars in high schools. During the 2017–2018 school year, the project reached over 20,800 students from 157 high schools through 202 seminars. Targeting education at an early age, independently of the stages of athletic careers (which can vary by sport), may positively influence doping prevention and should be implemented as early as possible [31,32]. Early anti-doping education is an essential pillar in the prevention of doping. It should encompass raising awareness, providing information, promoting values-based education, and delivering anti-doping education [33]. 

For adolescents, their concerns about body image represent the primary risk factor for using performance-enhancing substances [34,35]. Therefore, it is crucial to focus on improving young people’s and athletes’ awareness and understanding of the risks and consequences of doping in sports, helping to build a sense of responsibility together with elevated levels of self-esteem and reduced trait anxiety as they are protective factors against the use of doping substances [36,37,38,39]. This multidimensional approach can foster a strong ethical foundation in young people, equipping them with the knowledge and values necessary to make informed choices and resist the pressures to engage in doping behaviors.

## 2. The Project: “Sport Informa”

### 2.1. General Aims

The project aimed to strengthen the educational network to counteract the use of doping substances, with the goal of protecting health in sports activities. It sought to create an informed and prepared educational community on the topic of doping substances and protective factors, focusing on non-technical figures surrounding the world of sports who interact with young people during their developmental years, while also directly involving the youth. The general objectives of the project were as follows: (i) to strengthen the educational network to counteract the use of doping substances in order to protect health in sports activities; (ii) to create an informed and prepared educational community into the schools on the topic of doping substances and protective factors, involving non-technical figures from the world of sports who interact with young people in their developmental years and the youth themselves; and (iii) to increase awareness among the educational community and children and young people in their developmental years. These three activities were accompanied by a communication campaign and scientific research aimed at understanding the doping phenomenon from the perspective of both educators and young people. The project was implemented in the following Italian locations: Turin, Chieti, Rome, Latina, Caserta, Catania, and Cagliari. The project included different tasks such as the following:

(a) A training activity directed at teachers, educators, and coordinators of summer and winter camps to provide courses and tools designed to enhance self-esteem and self-management skills, with the objective of fostering balanced psychophysical development in young people to prevent the risk of doping.
(b) An awareness-raising activity targeting an age group of 11–19, through webinars and meetings with Olympic and Paralympic sports champions to discuss the risks of doping.
(c) A practical activity involving participation in multidisciplinary events for 11–19 age groups, supported by a communication campaign promoting doping prevention and healthy lifestyles.
(d) A survey was administered to adults who participated in the training and to the 14–19 age group to assess their knowledge of the risks associated with doping substances.
(e) A nationwide communication campaign aimed at broadening the reach of the project’s message.

### 2.2. Current Study 1: Teachers

#### 2.2.1. Method

This study takes into consideration the first task of the project. As outlined in the general objectives, the Sport Informa Project, conducted in November 2023, offered an online training program aimed at educators and teachers across all educational levels. The course provided participants with knowledge about the phenomenon of doping and introduced practical tools to enhance self-esteem and self-management skills. Upon completing the training, participants were encouraged to implement the acquired skills and tools in their daily interactions with young people.

To assess whether the training resulted in any significant changes, a longitudinal study was carried out during the 2023/2024 school years. Data collection occurred both before and after the training (pre- and post-training phases). Specifically, two periods were analyzed for each phase: the initial phase (November–December 2023) and the final phase (January–February 2024). This evaluation was conducted using specific psychometric instruments administered before and after the training activities: the Self-Efficacy Scale [40], the Self-Compassion Scale [41], and the Emotional Regulation Questionnaire (ERQ) [42].

#### 2.2.2. Sample

In this study, a non-probabilistic sampling method was employed, whereby participants were invited to take part in the study on a voluntary basis. As outlined in the general aims, the communication and awareness-raising campaign for the doping project was implemented in the schools of the following Italian locations: Turin, Chieti, Rome, Latina, Caserta, Catania, and Cagliari. Teachers and school principals were invited to participate in the planned training and related research activity consisting of the two phases (pre- and post-training).

The sample included 118 teachers, 59 in the first phase (pre-training) and 59 in the second phase (post-training), who were involved in the training activities.

The sample is mainly composed of women (78.0%) compared to men (22.0%). All interviewees have the role of teacher. The average age of the sample is 53.7 years, higher for women (54.3) than for men (51.3). 

The most common educational qualification is a Master’s degree (64.4% of the total), and 28.8% have a Bachelor’s Degree, while 6.8% have a high school diploma.

#### 2.2.3. Teacher Training

The 16 h training program, conducted by expert trainers, was delivered through the Niccolò Cusano University’s online platform and was designed for educators and teachers at all educational levels. The course was designed to raise awareness about the phenomenon of doping and to equip participants with tools to enhance self-esteem and self-management skills. The course focused on doping phenomenon; specifically, information about the nature of doping, its impact on physical and mental health, and its prevalence, and on psychological processes aimed at enhancing self-esteem, self-regulation, and self-compassion among young people. This was intended to help educators foster a supportive environment and address the psychological factors related to doping.

Therefore, the course covered a variety of topics, including the following: (i) the phenomenon of doping (its characteristics and prevalence in Italy), (ii) the impact on physical and mental health, (iii) risk and protective factors, and (iv) soft skills and tools to strengthen protective factors in young people.

#### 2.2.4. Data Collection

This study was conducted according to the guidelines of the Declaration of Helsinki and approved by the Institutional Review Board of Niccolò Cusano University. All participants signed the informant consent.

#### 2.2.5. Procedure

##### Tests

In order to obtain the planned data, the following instruments were employed as follows:

- Perceived Self-Efficacy in Managing Complex Problems Scale [40];
- Self-Compassion Scale [41];
- ERQ—Emotional Regulation Questionnaire [42].

These tests were implemented to assess the effectiveness of the training, particularly to measure the improvement in self-efficacy, self-compassion, and emotional regulation. All questionnaires were administered before and after the training, and the Google Forms platform was used for this purpose.

All data analyses were conducted using SPSS Statistics 29.0.1.0 and Jamovi (The Jamovi Project, 2024—Version 2.5, retrieved from https://www.jamovi.org, accessed on 1 May 2024).

#### 2.2.6. Psychometric Test: Perceived Self-Efficacy in Managing Complex Problems 

The concept of self-efficacy is typically examined in relation to specific tasks [43,44]. In this context, the focus is on teachers’ self-efficacy in raising awareness among young people about the risks associated with using performance-enhancing substances to counter harmful health behaviors. Research indicates that teachers’ self-efficacy significantly impacts various aspects of their professional practice [45]. Specifically, teachers with a high sense of self-efficacy are more adept at planning, organizing, and tailoring instructional activities to their students’ needs [46,47]. They are also more inclined to embrace innovative ideas and pedagogical approaches to enhance student learning [48,49]. Moreover, studies suggest that teachers with strong self-efficacy in managing challenging student behaviors experience lower stress levels. These teachers also implement more effective classroom management strategies that address student needs, fostering more positive behaviors in the classroom [50,51]. These traits are critical for the current research project, which seeks to raise awareness among young people about the dangers of performance-enhancing substance use and to encourage healthy behaviors in sports activities.

#### 2.2.7. Measure: Self-Efficacy Scale

To assess the perception of self-efficacy within the educational community involved in the project, the “Perceived Self-Efficacy Scale for Managing Complex Problems”, developed by Avallone and colleagues (2017) [40], was utilized.

This scale, employed in the analyzed questionnaire, includes 24 items divided into 4 dimensions (emotional maturity, goal-oriented action, relational fluidity, context analysis). Participants were asked to self-evaluate their capabilities using a Likert scale, where 1 corresponds to “not capable at all” and 5 to “fully capable”. Each dimension includes 6 of the 24 items, thus allowing for a minimum score of 6 (1 × 6) and a maximum score of 30 (5 × 6) for each dimension and each respondent.

The self-efficacy test showed good internal consistency for both phases (1st phase: Cronbach’s α = 0.90; 2nd phase: Cronbach’s α = 0.95). Furthermore, recalculating Cronbach’s α after removing an item confirms the level of consistency of the item complex as there are good correlation values [52] (r > 0.3) with the total score.

#### 2.2.8. Analysis

Data analysis for each dimension involved summing the scores of the respective items. A separate descriptive analysis was conducted for each dimension and survey phase. For each frequency distribution, positional indices (such as the mean) and dispersion indices (such as standard deviation and range) were calculated.

Furthermore, Table 1 displays the Skewness index (Skewness with respect to the mean) and the Kurtosis index, which were calculated to assess the consistency of the score distribution shape. The Shapiro–Wilk normality test was also performed.

A parametric repeated-measures ANOVA was conducted, and Cohen’s d index was calculated to evaluate the effect size (ES) (interpretation references: less than 0.05 = small effect, between 0.05 and 0.20 = medium effect, greater than or equal to 0.20 = large effect, as defined in Kraft (2020) [53] and (2023) [54]).

Finally, a factor analysis was performed to identify potential latent dimensions that could help summarize and explain the relationships between the original variables using new factors.

#### 2.2.9. Results

The Shapiro–Wilk normality test produced a non-significant result, indicating that the null hypothesis of normal distribution was accepted (Table 1). However, this was not the case for the 1st phase survey of the Context Analysis dimension, where the normality test returned a significant result, leading to the rejection of the null hypothesis of normal distribution. Nonetheless, for this dimension, the observed values of the Skewness and Kurtosis indices fell within the acceptable range of −1 to +1 [55].

A parametric ANOVA for repeated measures was conducted, as Levene’s test for the homogeneity of variance was not significant for all four dimensions of self-efficacy considered: Emotional Maturity (F(1, 116) = 1.84, *p* = 0.178); Goal-Oriented Action (F(1, 116) = 2.40, *p* = 0.124); Relational Fluidity (F(1, 116) = 1.61, *p* = 0.208); Context Analysis (F(1, 116) = 3.21, *p* = 0.076).

The results of the ANOVA indicated a significant increase (*p* < 0.05) in the mean values from the first to the second measurement for all the dimensions considered, with a large effect size (Kraft (2020) [53] and (2023) [54]): Emotional Maturity (F(1, 58) = 4.356, *p* = 0.041; d = 0.272); Goal-Oriented Action (F(1, 58) = 9.000, *p* = 0.004; d = 0.391); Relational Fluidity (F(1, 58) = 4.340, *p* = 0.042; d = 0.271); Context Analysis (F(1, 58) = 5.996, *p* = 0.017; d = 0.319).

**Figure 1:**
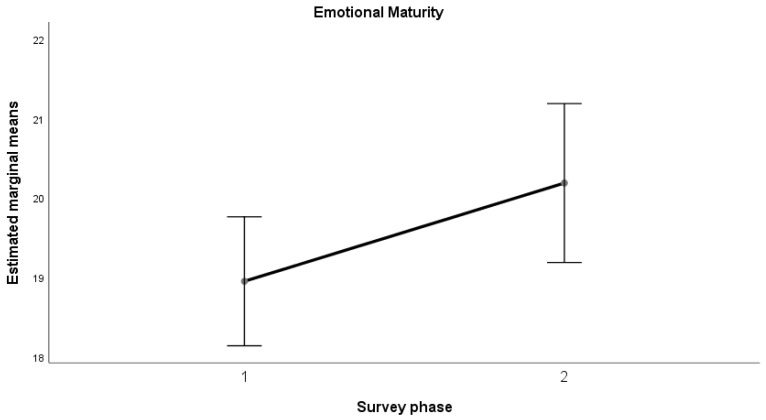
Emotional Maturity—average trend.

**Figure 2:**
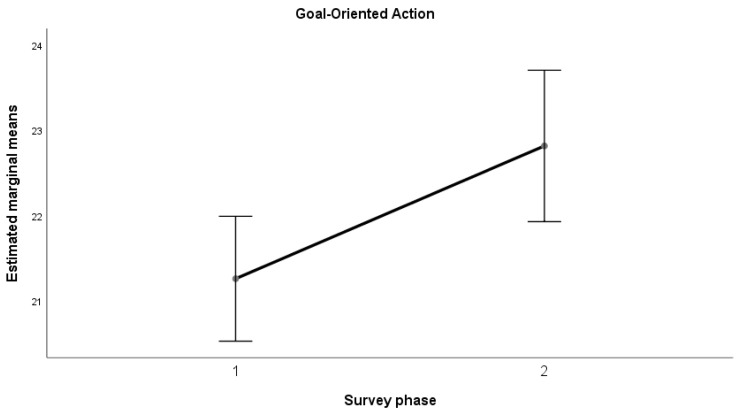
Goal-Oriented Action—average trend.

**Figure 3:**
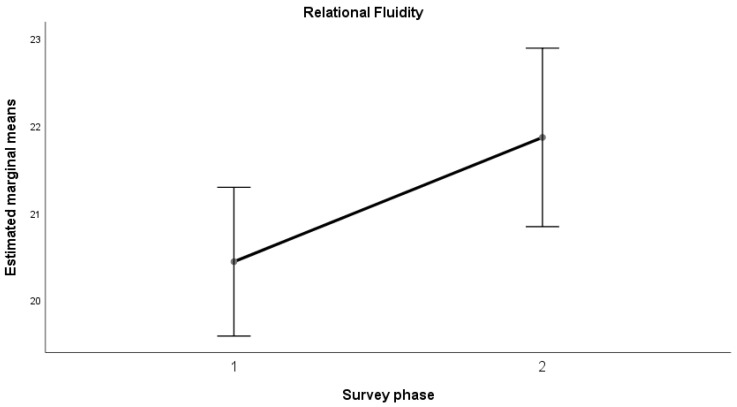
Relational Fluidity—average trend.

**Figure 4:**
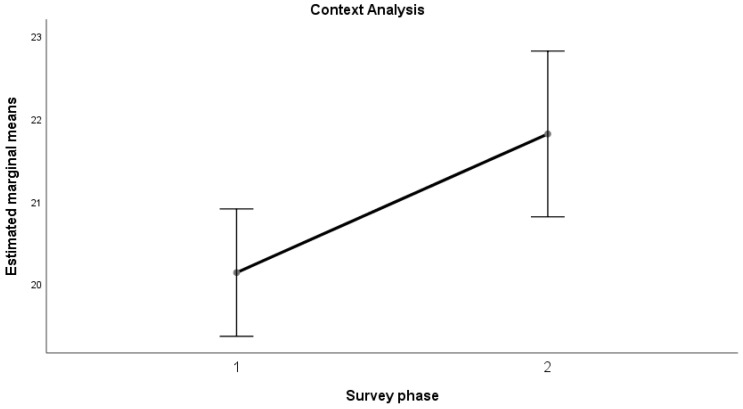
Context Analysis—average trend.

This trend can also be observed in the following graphs (Figure 1, Figure 2, Figure 3 and Figure 4).

Finally, a factor analysis was conducted to identify potential ‘latent dimensions’ that could help summarize and explain the relationships between the original variables with new factors [55]. The Kaiser–Meyer–Olkin (KMO) Test for Sampling Adequacy, and Bartlett’s Test of Sphericity indicate that it is appropriate to proceed with the factor analysis of the data (see Table 2).

The latent structure allowed the identification of three subdimensions within the set of values. The first area related to relationships and interactions with people, which includes the following items: 14. Engaging in discussions with others; 13. Easily joining a group; 17. Establishing good relationships with everyone; 18. Managing conflicts with people; 24. Interpreting the requests made by others; 15. Providing support to others. The second area related to commitments and the importance of setting and achieving proposed goals: 9. Achieving the goals you have set for yourself; 7. Setting concrete and achievable goals; 8. Setting goals to be achieved in the future; 12. Meeting deadlines; 10. Prioritizing tasks; 11. Identifying goals that match one’s capabilities. The third area related to the importance of understanding and adapting to various contexts: 19. Understanding the context in which I find myself; 22. Using appropriate language for different contexts and situations; 23. Understanding what others expect from you in different situations; 20. Comprehending the relationships between different facts and situations; 21. “Reading” the environment and situations from multiple perspectives. Factor analysis was useful for summarizing the available information and highlighting a different grouping of factors compared to the initial one. Internal consistency measures confirm that the constructed indices are reliable (Cronbach’s α = 0.921; 1st phase: α = 0.895; 2nd phase α = 0.931). 

## 3. Psychometric Test: Self-Compassion Scale

Self-compassion involves being open and sensitive to one’s own suffering, accompanied by feelings of care and kindness toward oneself. It entails adopting a non-judgmental and understanding attitude toward one’s inadequacies and failures, recognizing that one’s experiences are part of the common human experience [56]. It enables individuals to address external challenges or stressors, such as societal or peer pressure regarding appearance, with a calm, non-reactive, and non-judgmental mindset. Additionally, self-compassion is recognized for its role in reducing tendencies toward excessive self-focus and harsh self-criticism [56]. In the context of body image and performance, self-compassion offers a valuable framework for interrupting the psychological processes that contribute to the development of low self-esteem related to body image and performance.

The concept of self-compassion includes various aspects, as indicated by Neff (2003) [56]: having kindness and understanding toward oneself rather than resorting to harsh self-criticism and judgment; viewing one’s experiences as part of a larger human context, rather than as isolated and separate events; and maintaining a balanced awareness of one’s painful thoughts and feelings, avoiding over-identification with them. 

### 3.1. Measure: Self-Compassion Scale

To measure self-compassion, Neff (2003) [56] developed the Self-Compassion Scale, and in this study, the Italian version proposed by Veneziani et al. (2017) [41] was implemented. The scale is composed of six constructs (26 items), each comprising one positive and one oppositional dimension: (1a) Self-Kindness (item 5-12-19-23-26), in terms of kindness and tolerance of one’s own limits and inadequacies, (1b) Self-judgment (item 1-8-11-16-21), on the opposite, as a negative judgment and intolerance towards one’s own weaknesses; (2a) Common Humanity (items 3-7-10-15), as a sense of sharing one’s limitations and weaknesses with the rest of humanity, (2b) Isolation (items 4-13-18-25), on the opposite, as a sense of isolation and separation from others; (3a) Mindfulness (items 9-14-17-22), as a positive mobilization of one’s feelings and emotions in the face of difficulties; (3b) Over-Identification (items 2-6-20-24), on the opposite, as self-pity and focusing on one’s negative feelings.

Participants were asked to self-evaluate using a Likert scale, where 1 corresponds to “almost never” and 5 to “almost always”. The result for each dimension is the mean of the scores obtained from the corresponding items. In the statistical elaborations, the scale scores of opposition dimensions were inverted (Self-Judgment; Isolation; Over-Identification).

The self-compassion test showed good internal consistency for both phases (1st phase: Cronbach’s α = 0.88; 2nd phase: Cronbach’s α = 0.87). Furthermore, recalculating Cronbach’s α after removing an item confirms the level of consistency of the item complex as there are good correlation values [52] (r > 0.3) with the total score.

### 3.2. Analysis

The data analysis for each dimension referred to the mean of the scores of the respective items, and a separate descriptive analysis was carried out in both dimension and survey phases. For each frequency distribution, position indices, such as mean, as well as dispersion indices, such as standard deviation and range, were calculated. Furthermore, Table 3 shows the Skewness index (Skewness with respect to the mean) and the Kurtosis index calculated to verify the consistency of the shape of the score distribution, and the Shapiro–Wilk normality distribution test. A paired sample *t*-test was performed to assess the variation in mean scores, and the Cohen’s d index was calculated to evaluate the Effect Size (ES) (interpretation references: less than 0.05 is a small effect, between 0.05 and 0.20 is a medium effect, and more or equal to 0.20 is a large or greater effect, in Kraft (2020) [53] and (2023) [54]). 

### 3.3. Results

The Shapiro–Wilk normality test yielded statistically significant results (*p* < 0.05), indicating that the null hypothesis of normality of distribution was rejected for the Self-Kindness, Common Humanity, Isolation, and Mindfulness variables in the 1st phase, as well as for the Self-Kindness and Isolation variables in the 2nd phase (Table 3). In contrast, the normality test yielded non-significant results for the remaining dimensions and phases. For each dimension, the observed values of the Skewness and Kurtosis indices are within the acceptable range of −1 to +1 [55].

In order to perform a Student’s *t*-test to assess the potential significance of the averages, the homogeneity of variance test (Levene) was also carried out, which was found to be non-significant for all six dimensions of self-compassion under consideration. Self-Kindness (F(1, 116) = 0.021, *p* = 0.886); Self-Judgment (F(1, 116) = 0.148, *p* = 0.702); Common Humanity (F(1, 116) = 3.003, *p* = 0.086); Isolation (F(1, 116) = 1.204, *p* = 0.275); Mindfulness (F(1, 116) = 2.629, *p* = 0.108); and Over-Identification (F(1, 116) = 1.517, *p* = 0.221).

A paired-sample *t*-test was performed to assess a possible increase in the mean scores from the 1st to the 2nd phase for the dimensions of Self-Kindness, Common Humanity, and Mindfulness. For the dimensions of Self-Judgment, Isolation, and Over-Identification, the goal was to evaluate a possible decrease in the mean values between the 1st and 2nd phases. No significant increase was observed for Self-Kindness with an average effect size (t(58) = 1.447; *p* = 0.153, d = 0.188) and for Mindfulness with a large effect size (t(58) = 1.821; *p* = 0.074, d = 0.237). However, the increase was significant for Common Humanity with a large effect size (t(58) = 3.246; *p* = 0.002, d = 0.423). Regarding the “opposing” dimensions, the comparison was considered in terms of a decrease, calculating the difference between the 1st and 2nd phases under the hypothesis that the data from the 1st phase would be greater than those from the 2nd phase. The differences were found to be insignificant for all three dimensions (Self-Judgment, Isolation, Over-Identification) with a moderate effect size (Table 3). 

### 3.4. Psychometric Test: ERQ—Emotional Regulation Questionnaire

The Emotional Regulation Questionnaire (ERQ) is a questionnaire designed to measure the tendency to regulate one’s emotions through two emotion regulation strategies: Cognitive Reappraisal and Expressive Suppression. Dysfunctional behaviors such as doping and substance abuse are negatively correlated with self-regulatory failure [57]. The ability to regulate their own behavior plays a crucial role in determining the likelihood of engaging in doping; specifically, high self-regulatory efficacy directly reduces the likelihood of using banned performance-enhancing substances by empowering individuals to resist temptations and make ethical decisions under pressure. Self-regulatory efficacy also indirectly affects doping behavior by reducing moral disengagement [58]. 

### 3.5. Measure: ERQ-Scale

In this study, the Italian validation of the instrument by Balzarotti (2010) [42] was used. The questionnaire consists of 10 items, with six assessing Cognitive Reappraisal (item 1-3-5-7-8-10), and the remaining four focused on Expressive Suppression (item 2-4-6-9).

Data are collected using a 7-point Likert scale (1 = strongly disagree; 4 = neutral; 7 = strongly agree). The total score of the questionnaire is calculated separately by summing the scores of the items for the two dimensions; thus, for each respondent, for the Cognitive Reappraisal dimension, which includes six items, the minimum score will be 6 (1 × 6) and the maximum score will be 42 (7 × 6), while for the Expressive Suppression dimension, which includes four items, the minimum score will be 4 (1 × 4) and the maximum score will be 28 (7 × 4). 

To understand the ability to regulate one’s emotions in a more functional manner, it is possible to consider the potential variation in the scores obtained from the first and second phases of the test administration. In particular, it would be expected that a higher mean score would be obtained from the scale concerning Cognitive Reappraisal, and a lower mean score would be obtained from the scale concerning Expressive Suppression.

The ERQ test showed an acceptable internal consistency for both phases (1st phase: Cronbach’s α = 0.72; 2nd phase: Cronbach’s α = 0.66). (The acceptability of the Cronbach’s alpha value takes into account the sample size of each phase n = 59, and the overall number of items, which is 10; [59].) Furthermore, recalculating Cronbach’s α after removing an item confirms the level of consistency of the item complex as there are good correlation values [52] (r > 0.3) with the total score.

### 3.6. Analysis

The data analysis for each dimension referred to the sum of the scores of the respective items, and a separate descriptive analysis was carried out for both the dimension and survey phase. For each frequency distribution, position indices, such as mean, as well as dispersion indices, such as standard deviation and range, were calculated. Furthermore, Table 4 shows the Skewness index (Skewness with respect to the mean) and the Kurtosis index calculated to verify the consistency of the shape of the score distribution, and the Shapiro–Wilk normality distribution test. A parametric ANOVA for repeated measures was conducted, and the Cohen’s d index was calculated to evaluate the Effect Size (ES) (interpretation references: less than 0.05 is a small effect, between 0.05 and 0.20 is a medium effect, more or equal to 0.20 is a large or greater effect, in Kraft (2020) [53] and (2023) [54]). 

### 3.7. Results

The Shapiro–Wilk normality test yielded statistically non-significant results, indicating that the null hypothesis of normality of distribution was accepted (Table 4). For each dimension, the observed values of the Skewness and Kurtosis indices are within the acceptable range of −1 to +1 [55].

A parametric ANOVA for repeated measures was conducted, as the Levene test for verifying the homogeneity of variance was not significant for both dimensions of the ERQ scale that were considered, as follows: Cognitive Reappraisal F(1, 116) = 2.31, *p* = 0.131, and Expressive Suppression F(1, 116) = 1.82 × 10^−30^, *p* = 1.000. The ANOVA results revealed a significant difference (*p* < 0.001) between the mean value of Cognitive Reappraisal and Expressive Suppression, with a large effect size F(1, 116) = 467.24; ƞ = 0.654. Post hoc tests confirmed the significant difference between the two phases for both dimensions considered (1st Phase Cognitive Reappraisal vs. 1st Phase Expressive Suppression: t(58) = 14.394, *p* < 0.001; 1st Phase Cognitive Reappraisal vs. 1st Phase Expressive Suppression: t(58) = 14.746, *p* < 0.001), while no significant differences were found between the mean scores of the 1st and 2nd phases for Cognitive Reappraisal (t(58) = 1.415, *p* = 0.163) or for Expressive Suppression (t(58) = 0.0629, *p* = 0.95).

**Figure 5:**
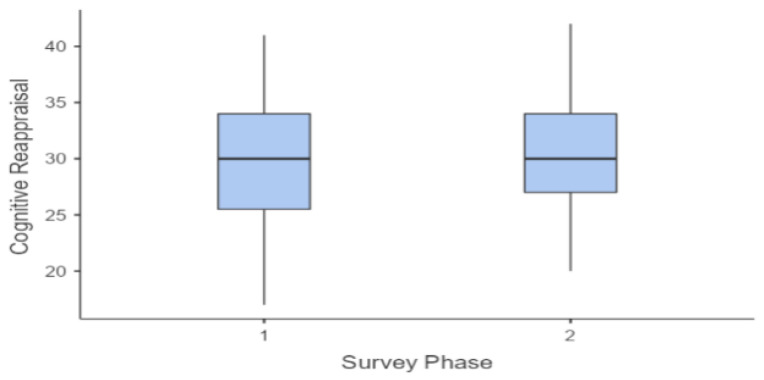
Cognitive Reappraisal—average trend.

**Figure 6:**
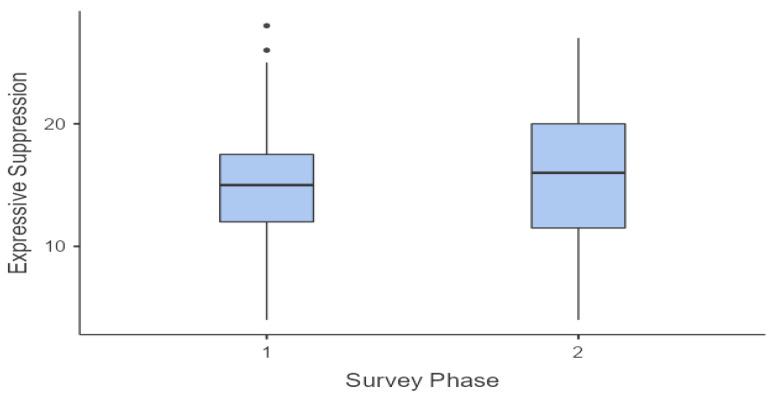
Expressive Suppression—average trend.

This trend can also be observed in the following graphs (Figure 5 and Figure 6).

## 4. Explorative Study on Students 

### 4.1. Method

In addition to the training activity and the research carried out for teachers and educators, in May 2024, an explorative survey was conducted among students of educators who had participated in the various activities envisaged by the project and described in the general aims. The purpose of the survey was to determine the initial self-assessment levels of specific psychological constructs relevant to doping prevention. These findings would serve as a foundation for designing future educational interventions and planning events aimed at fostering protective factors against doping.

Considering what the scientific literature has shown, i.e., that high levels of self-esteem and low levels of trait anxiety can protect against the use of doping substances [36,37], a survey was carried out by administering the specific psychometric test, as the Multidimensional Test on Self-Esteem (TMA) [60,61].

### 4.2. Sample

In this study, a non-probabilistic sampling method was employed, whereby participants were invited to participate voluntarily. As outlined in the general aims, the communication and awareness-raising campaign for the doping project was implemented in the schools of the following Italian locations: Turin, Chieti, Rome, Latina, Caserta, Catania, and Cagliari.

The survey was conducted on a sample of 124 young people aged 14–19, who participated in the activities planned by the project, through the administration of a questionnaire.

The sample involved boys (62.1%) and girls (37.9%), and is mainly composed of students that are 16 years old (43.5%), followed by 15 years old (33.9%). The sports high school is the most attended (29.8%). 

### 4.3. Measures

The Multidimensional Self-Esteem Test [60,61] was utilized to measure students’ self-esteem. The questionnaire was administered using the Google Forms platform.

### 4.4. Psychometric Test: The Self-Esteem Scale

The Multidimensional Self-Esteem Test [60] is based on the multidimensional conception of self-esteem. According to Shavelson et al. (1976) [62], on which Bracken’s model (1992) [60] is based, the self-concept can vary depending on different areas of experience and social demands. Therefore, it can be defined as a multidimensional construct. The various dimensions of the self-concepts are hierarchically organized and structured, and they influence overall levels of self-esteem.

### 4.5. Measure: The Self-Esteem Scale

To assess the perception of self-esteem within the students involved in the project, the “Multidimensional Self-Esteem Test” [60] was utilized.

This scale, used in the analyzed questionnaire, consists of 150 items divided into six dimensions (25 items for each dimension): Interpersonal relations, Competence in Environmental Control, Emotionality, Academic Success, Family Life, and Physical Self.

The items, even within each dimension, can have a positive or negative connotation. The Likert scale used to calculate scores with a positive connotation is as follows: absolutely true = 4; true = 3; not true = 2; absolutely not true = 1. If it has a negative connotation, it is as follows: absolutely true = 1; true = 2; not true = 3; absolutely not true = 4.

For each dimension, the raw score is calculated as the sum of the values obtained from the respective items. The overall self-esteem score is derived from the sum of all raw values across six scales. In order to provide an interpretation of the results, the raw scores are transformed into standard scores based on normative tables provided by Bracken (2003) [63]. Subsequently, for each of the six specific areas and for the total, the self-esteem score is calculated and divided into seven ranges (from extremely positive to extremely negative self-esteem).

The TMA test showed a good internal consistency of raw scores (Cronbach’s α = 0.78). Furthermore, recalculating Cronbach’s α after removing an item confirms the level of consistency of the item complex as there are good correlation values [52] (r > 0.3) with the total score. The gender-specific analysis confirms the evidence of the scale described.

### 4.6. Analysis

Standard scores were calculated by transforming the raw scores on the basis of the normative data published by the author, and the data analysis for each dimension and gender referred to the sum of the scores of the respective items. For each frequency distribution, position indices, such as mean, as well as dispersion indices, such as standard deviation and range, were calculated. Furthermore, Table 5 shows the Skewness index (Skewness with respect to the mean) and the Kurtosis index calculated to verify the consistency of the shape of the score distribution, and the Shapiro–Wilk normality distribution test. A sample *t*-test was performed to assess the variation in mean scores between genders, and the Cohen’s d index was calculated to evaluate the Effect Size (ES) (interpretation references: less than 0.05 is a small effect, between 0.05 and 0.20 is a medium effect, more or equal to 0.20 is a large or greater effect, in Kraft (2020) [53] and (2023) [54]). 

Finally, the percentages of male and female students in the self-esteem ranges identified by the model were calculated according to the classification in Table 6. 

## 5. Results

The Shapiro–Wilk normality test yielded statistically significant results (*p* < 0.001), indicating that the null hypothesis of normality of distribution was rejected for the following dimensions: Interpersonal Relationships (overall sample and gender); Academic Success (overall sample and male, female *p* <  0.05); and Family Life and Global Self-Esteem (overall sample and male). In contrast, the normality test yielded non-significant results for the remaining dimensions. For each dimension, the observed values of the Skewness and Kurtosis indices are within the acceptable range of −1 to +1 [55].

In order to perform a Student’s *t*-test to assess the average significance by gender, the homogeneity of variance test (Levene) was also carried out, which was found to be non-significant for all six dimensions of the TMA test under consideration: Interpersonal Relationships (F(1, 122) = 0.421, *p* = 0.518); Competence in Environmental Control (F(1, 122) = 1.207, *p* = 0.274); Emotionality (F(1, 122) = 0.338, *p* = 0.562); Academic Success (F(1, 122) = 0.384, *p* = 0.537); Family Life (F(1, 122) = 3.719, *p* = 0.056); and Physical Self (F(1, 122) = 0.740, *p* = 0.391).

Independent sample *t*-tests were performed to evaluate the possible difference for each dimension obtained by boys compared to girls. Results showed no significant differences for each dimension of the TMA test; only for the Academic Success dimension was the negative t-value recorded due to a higher mean score for girls compared to boys. Therefore, the slightly higher mean value for boys than for girls observed for each dimension is consistent with the findings in the literature. Specifically, large effect sizes (Cohen’s d) were observed for Competence in Environmental Control (t(122) = 2.115; *p* = 0.036, d = 0.3915), Family Life (t(122) = 1.719; *p* = 0.088, d = 0.3181), and Academic Success (t(122) = −1.248; *p* = 0.215, d = 0.2309); medium effect sizes (Cohen’s d) for Interpersonal Relationships (t(122) = 0.543; *p* = 0.588, d = 0.1005) and Emotionality (t(122) = 0.749; *p* = 0.455, d = 0.1387); a small effect size (Cohen’s d) for Physical Self (t(122) = 0.222; *p* = 0.825, d = 0.0410); the Global Self-Esteem value shows a large effect size (t(122) = 1.383; *p* = 0.169, d = 0.2561). 

Finally, Table 6 shows the percentages of male and female students in the self-esteem ranges identified and calculated according to the classification [63].

In the highest estimation ranges (standard score above 115: from slightly positive to extremely positive self-esteem), students are absent on all dimensions analyzed.

**Figure 7:**
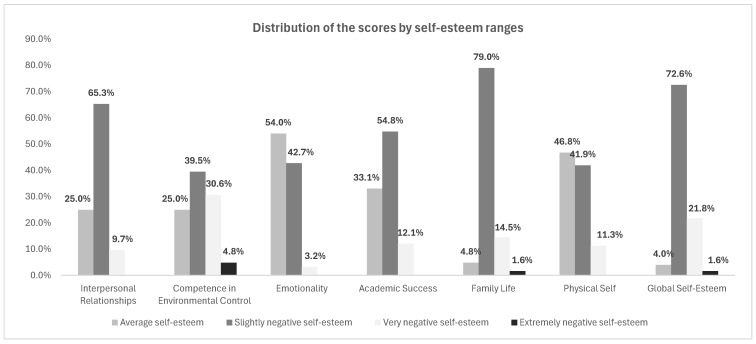
TMA dimensions—classification range score.

For the Interpersonal Relationships, Emotionality, Academic Success, and Physical Self dimensions, the percentages are concentrated in the ranges with scores between 66 and 115 (very negative, slightly negative, and average self-esteem), while Competence in Environmental Control and Life Family dimensions shows slight values in the score range below 66 (extremely negative self-esteem). In the slightly negative self-esteem range, the highest percentages were observed in the Family Life (79.0%), Interpersonal Relationships (65.3%), and Academic Success (54.8%) dimensions. In the medium self-esteem range, the highest percentages were observed in the Emotionality (54.0%) and Physical Self (46.8%) dimensions. Figure 7 shows the total scores for each dimension, while the values broken down by gender are shown in Table 6.

## 6. Discussion

Doping, particularly in recreational sports, is increasingly recognized as a public health concern. While doping in professional sports is banned and can lead to penalties as well as serious health risks for athletes, the issue extends beyond competitive sports [64]. The focus has shifted to non-athletic youth groups, reflecting the understanding that doping prevention is not only a matter for athletes but a broader societal issue [1]. Education that emphasizes the advantages of remaining substance-free plays a crucial role in protecting young people from the temptation of performance-enhancing substances. This approach aligns with the view that doping prevention should target society at large, not just those involved in professional or competitive sports [17]. 

Recent interventions concentrated both on raising awareness about banned substances and the dangers they are, and they have incorporated knowledge about psychological aspects that play a role in doping behavior [65]. For example, Barkoukis et al. (2016) [39] aimed to raise awareness among participants about the psychological factors that contribute to doping, as well as the moral principles tied to sports ethics, such as fair play and sportsmanship. Similarly, Sagoe et al. (2016) [66] focused on educating participants about the ethical implications of doping and provided practical strategies to help them resist peer pressure to engage in such behavior. Lucidi et al. (2017) [67] and Mallia et al. (2020) [68] implemented interventions centered on media literacy, teaching participants to critically analyze how media messages often minimize or ignore the moral issues associated with doping and how such messages can influence perceptions and decisions. 

An important aspect to consider is the emotion, which is a powerful motivator of behavior, that is often overlooked by theoretical approaches to doping [38,65]. In this line, it is crucial to consider the concept of self-regulation which refers to an individual’s perceived ability to manage or overcome specific situations or challenges that could pose risks to their well-being. This concept is significant because it shapes behavior by impacting resilience in difficult circumstances, susceptibility to social pressure, and the choices and actions individuals take [69]. Findings from research indicate that athletes who have a stronger ability to self-regulate—specifically, to resist the temptation to use performance-enhancing substances—are less likely to harbor intentions to dope and less likely to engage in doping behaviors. This inverse relationship has been supported by studies, including those conducted by Barkoukis et al. (2016) [39], Lucidi et al. (2008) [21], and Ring et al. (2019) [70]. In addition, research highlights the link between self-compassion and psychological processes in the context of sports. Mosewich and colleagues (2011) [71] showed that higher levels of self-compassion were associated with lower levels of shame proneness, guiltless shame proneness, anxiety about social physique, objectified body awareness, fear of failure, and fear of negative judgment from others in athletes. These findings suggest that self-compassion may serve as a valuable tool in managing maladaptive self-conscious emotions, self-critical thoughts, and behaviors tied to both appearance and performance. 

The Italian project “Sport Informa” aimed to strengthen the educational network to counteract the use of doping substances, with the goal of protecting health in sports activities. The study investigates the impact of a training intervention for educators and teachers on self-efficacy, self-compassion, and emotional regulation in the context of doping prevention among young people. The research aimed to (i) equip educators and teachers with tools to raise awareness about the dangers of doping and its impact on young people’s health; and (ii) promote the development of self-esteem and self-regulation in students to prevent doping-related behaviors by equipping teachers with strategies and tools to improve young people’s self-efficacy, emotional control, and self-management skills. 

Results revealed a positive trend across all psychometric scales used in the study, from self-efficacy to self-compassion and emotional regulation, with significant improvements in average scores between the pre- and post-training phases for teachers in the sample. In the self-efficacy scale, the dimensions of emotional maturity, goal orientation, relational fluidity, and contextual analysis showed significant increases, accompanied by large effect sizes. Similarly, data collected on teachers indicated a notable improvement in the self-compassion scale, particularly in the “Common Humanity” dimension, with a large effect size. This in turn equipped them with the tools to positively influence their students, promoting healthier behaviors and a better understanding of the risks associated with doping. While there was no significant change in “Self-Kindness” and “Mindfulness,” the opposite dimensions (Self-Judgment, Isolation, Over-identification) showed a non-significant decrease with moderate effect sizes. Lastly, the Emotional Regulation Questionnaire (ERQ) demonstrated a significant difference between the averages for cognitive reappraisal and expressive suppression, with a large effect size. The results highlight the positive and significant impact of the training activities in strengthening psychological dimensions that serve as key protective factors against harmful and unhealthy behaviors. This aligns with the existing literature, which demonstrates that the level of autonomous motivation in teenage athletes is a crucial predictor of their sportsmanship behaviors during games [72]. However, the absence of a pre-test assessment for students before the teachers’ training limits the ability to measure the indirect effects of the intervention on students. Specifically, it remains unclear how much the application of the training tools by teachers enhanced students’ psychological resilience and awareness. Addressing this limitation could represent an important direction for future research.

## 7. Limitations and Future Directions

This study presents diverse limitations. First of all, the numerosity of the sample was limited, thus a study with a larger sample should be preferred. In addition, the study focuses on pre- and post-intervention evaluations on teachers within a limited timeframe. Long-term effects of the intervention on both educators’ practices might not have been adequately captured. A longer time frame and follow-up assessments would be desirable to understand the long-term effects of the interventions. In addition, a pre-test assessment for students is crucial; while teachers received training and implemented strategies, direct measures of students’ engagement with these interventions have not been explored, making it harder to evaluate how students experienced or benefited from the tools. Finally, it could be useful also to explore additional psychological constructs, such as resilience, empathy, or social connectedness, since this could provide a more holistic understanding of how interventions influence doping prevention. 

## Figures and Tables

**Table 1:** Self-efficacy dimensions—descriptive statistics.

	Survey Phase	N	M	SD	Min	Max	Skewness	Kurtosis	Shapiro-Wilk	
									W	*p*
Emotional Maturity (item 1–6)	1	59	18.9	3.12	9	25	−0.72	0.831	0.962	0.063
	2	59	20.2	3.84	12	30	0.517	0.475	0.966	0.094
Goal-Oriented Action (item 7–12)	1	59	21.3	2.81	15	28	0.356	−0.328	0.947	0.012
	2	59	22.8	3.41	17	30	0.335	−0.695	0.957	0.037
Relational Fluidity (item 13–18)	1	59	20.4	3.27	15	28	0.253	−0.934	0.950	0.017
	2	59	21.9	3.92	14	30	0.183	−0.747	0.976	0.298
Context Analysis (item 19–24)	1	59	20.1	2.96	16	29	0.982	0.561	0.894	<0.001
	2	59	21.8	3.85	14	30	0.455	−0.281	0.958	0.038

For each dimension reference is made to the sum of the scores of the respective items.

**Table 2:** Bartlett and Kaiser–Meyer–Olkin tests.

Kaiser-Meyer-Olkin (KMO) Test for Sampling Adequacy		0.867
Bartlett’s test of Sphericity	χ²	1863.27
	df	276
	Sign.	0.000

**Table 3:** Self-Compassion dimensions—descriptive statistics.

	Survey Phase	N	M	SD	Min	Max	Skewness	Kurtosis	Shapiro-Wilk		t	df	*p*	Effect Size Cohen’s d
									W	*p*				
Self-Kindness (item 5, 12, 19, 23, 26)	1	59	3.22	0.756	1.00	4.40	−0.7588	0.5310	0.940	0.006	1.447	58	0.153	0.188
	2	59	3.33	0.807	1.00	5.00	−0.3233	0.7056	0.946	0.011				
Self-Judgment (item 1, 8, 11, 16, 21)	1	59	3.04	0.713	1.00	4.60	−0.5173	0.3566	0.964	0.079	0.388	58	0.700	0.051
	2	59	2.92	0.668	1.20	4.40	−0.1481	0.1892	0.981	0.468				
Common Humanity (item 3, 7, 10, 15)	1	59	2.96	0.690	1.75	4.25	−0.1024	−1.0081	0.955	0.029	3.246	58	0.002	0.423
	2	59	3.33	0.594	1.75	4.50	−0.0704	−0.1463	0.975	0.258				
Isolation (item 4, 13, 18, 25)	1	59	3.54	0.922	1.00	5.00	−0.3744	−0.2080	0.956	0.031	0.870	58	0.388	0.113
	2	59	3.44	0.806	1.75	5.00	0.3530	−0.5891	0.955	0.030				
Mindfulness (item 9, 14, 17, 22)	1	59	3.42	0.761	1.75	5.00	−0.2981	−0.4707	0.955	0.030	1.821	58	0.074	0.237
	2	59	3.57	0.646	1.75	5.00	0.1729	0.1833	0.961	0.059				
Over-identification (item 2, 6, 20, 24)	1	59	3.33	0.819	1.00	4.75	−0.2544	−0.0806	0.973	0.210	1.556	58	0.125	0.203
	2	59	3.20	0.731	1.50	5.00	−0.0280	0.1750	0.977	0.315				

For each dimension, the average score values of the respective items are given. Scale scores were inverted for the following dimensions: Self-Judgment; Isolation; Over-Identification.

**Table 4:** ERQ dimensions—descriptive statistics.

	Survey Phase	N	M	SD	Min	Max	Skewness	Kurtosis	Shapiro-Wilk	
									W	*p*
Cognitive Reappraisal	1	59	29.9	6.00	17	41	−0.202	−0.589	0.977	0.329
Expressive Suppression	2	59	30.8	4.95	20	42	0.309	−0.572	0.972	0.200
	1	59	15.5	5.30	4	28	0.195	0.121	0.970	0.150
	2	59	15.9	5.11	4	27	−0.114	−0.236	0.984	0.628

For each dimension reference is made to the sum of the scores of the respective items.

**Table 5:** TMA dimensions—descriptive statistics.

Dimensions	Sample Group	N	M	SD	Min	Max	Skewness	Kurtosis	W	*p*	t	df	*p*	Effect Size Cohen’s d
Interpersonal Relationships	F	47	82.4	7.46	70	103	1.222	1.707	0.886	<0.001	0.543	122	0.588	0.1005
	M	77	83.1	6.29	70	102	0.991	1.627	0.93	<0.001				
	Totale	124	82.8	6.73	70	103	1.073	1.577	0.915	<0.001				
Competence in Environmental Control	F	47	77.1	7.51	57	94	−0.248	0.289	0.984	0.758	2.115	122	0.036	0.3915
	M	77	80.4	8.56	57	99	−0.062	−0.112	0.991	0.857				
	Totale	124	79.1	8.29	57	99	−0.042	0.024	0.991	0.614				
Emotionality	F	47	86,0	5.7	75	103	0.811	1.147	0.954	0.064	0.749	122	0.455	0.1387
	M	77	86.8	5.93	75	103	0.187	−0.124	0.983	0.404				
	Totale	124	86.5	5.83	75	103	0.404	0.168	0.979	0.046				
Academic Success	F	47	85.2	8.65	70	113	1.299	2.206	0.900	<0.001	−1.248	122	0.215	0.2309
	M	77	83.3	7.63	70	113	1.004	2.033	0.943	0.002				
	Totale	124	84.0	8.05	70	113	1.152	2.152	0.926	<0.001				
Family Life	F	47	78.3	4.9	63	88	−0.388	0.821	0.967	0.201	1.719	122	0.088	0.3181
	M	77	79.6	3.82	63	88	−1.296	3.993	0.918	<0.001				
	Totale	124	79.1	4.29	63	88	−0.883	2.011	0.948	<0.001				
Physical Self	F	47	85.1	7.51	72	103	0.186	−0.396	0.975	0.407	0.222	122	0.825	0.0410
	M	77	85.4	6.71	72	101	−1.070	−0.262	0.977	0.183				
	Totale	124	85.3	6.99	72	103	0.019	−0.346	0.979	0.049				
Global Self-Esteem	F	47	77.6	4.74	63	87	−0.263	1.005	0.961	0.116	1.383	122	0.169	0.2561
	M	77	78.7	3.72	63	86	−0.916	3.271	0.932	<0.001				
	Totale	124	78.3	4.15	63	87	−0.637	1.911	0.954	<0.001				

For each dimension reference is made to the sum of the scores of the respective items.

**Table 6:** TMA dimensions—classification range score by gender.

Dimensions		Range Score	>135	126–135	116–125	86–115	76–85	66–75	<66
		Classification	Extremely Positive Self-Esteem	Very Positive Self-Esteem	Slightly Positive Self-Esteem	Average Self-Esteem	Slightly Negative Self-Esteem	Very Negative Self-Esteem	Extremely Negative Self-Esteem
Interpersonal Relationships	F		-	-	-	10	29	8	-
			-	-	-	21.3%	61.7%	17.0%	-
	M		-	-	-	21	52	4	-
			-	-	-	27.3%	67.5%	5.2%	-
	Totale		-	-	-	31	81	12	-
			-	-	-	25.0%	65.3%	9.7%	-
Competence in Environmental Control	F		-	-	-	6	21	17	3
			-	-	-	12.8%	44.7%	36.2%	6.4%
	M		-	-	-	25	28	21	3
			-	-	-	32.5%	36.4%	27.3%	3.9%
	Totale		-	-	-	31	49	38	6
			-	-	-	25.0%	39.5%	30.6%	4.8%
Emotionality	F		-	-	-	22	24	1	-
			-	-	-	46.8%	51.1%	2.1%	-
	M		-	-	-	45	29	3	-
			-	-	-	58.4%	37.7%	3.9%	-
	Totale		-	-	-	67	53	4	-
			-	-	-	54.0%	42.7%	3.2%	-
Academic Success	F		-	-	-	16	27	4	-
			-	-	-	34.0%	57.4%	8.5%	-
	M		-	-	-	25	41	11	-
			-	-	-	32.5%	53.2%	14.3%	-
	Totale		-	-	-	41	68	15	-
			-	-	-	33.1%	54.8%	12.1%	-
Family Life	F		-	-	-	4	31	11	1
			-	-	-	8.5%	66.0%	23.4%	2.1%
	M		-	-	-	2	67	7	1
			-	-	-	2.6%	87.0%	9.1%	1.3%
	Totale		-	-	-	6	98	18	2
			-	-	-	4.8%	79.0%	14.5%	1.6%
Physical Self	F		-	-	-	20	21	6	-
			-	-	-	42.6%	44.7%	12.8%	-
	M		-	-	-	38	31	8	-
			-	-	-	49.4%	40.3%	10.4%	-
	Totale		-	-	-	58	52	14	-
			-	-	-	46.8%	41.9%	11.3%	-
Global Self-Esteem	F		-	-	-	3	28	15	1
			-	-	-	6.4%	59.6%	31.9%	2.1%
	M		-	-	-	2	62	12	1
			-	-	-	2.6%	80.5%	15.6%	1.3%
	Totale		-	-	-	5	90	27	2
			-	-	-	4.0%	72.6%	21.8%	1.6%

## Data Availability

Data are unavailable due to privacy reasons. If necessary, you may contact the authors to request access.
